# Mental Health and Wellbeing of Population with Migrant Background in Switzerland – a Scoping Review and Evidence Map of Quantitative Evidence

**DOI:** 10.1007/s10903-023-01490-5

**Published:** 2023-05-26

**Authors:** Dawid Gondek, Laura Bernardi

**Affiliations:** Swiss Centre of Expertise in Life Course Research (LIVES), University of Lausanne, Lausanne, Switzerland

**Keywords:** Migrants, Refugees, Mental health, Wellbeing, Switzerland, Population

## Abstract

We aimed to map the evidence, based on population-based and migrant-specific datasets in Switzerland, on mental health and wellbeing of the population with migrant background. The research questions were: What is known from the existing quantitative evidence about the mental health of the population with migrant background living in Switzerland? What are the research gaps that can be addressed with existing secondary datasets in Switzerland? We used the scoping review approach to describe existing research. We searched Ovid MEDLINE and APA PsycInfo (2015 - September 2022). This resulted in a total of 1862 potentially relevant studies. In addition, we manually searched other sources, such as Google Scholar. We used a evidence map to visually summarise research characteristics and identify research gaps. In total, 46 studies were included in this review. Most studies used cross-sectional design (78.3%, n = 36) and theirs aims were descriptive (84.8%, n = 39). The studies tend to examine mental health or wellbeing of the population with migrant background in the context of social determinants (69.6%, n = 32). The most frequently studied social determinants were at the individual level (96.9%, n = 31). Out of 46 included studies, 32.6% (n = 15) included depression or anxiety, and 21.7% (n = 10) post-traumatic stress disorder and other traumas. Other outcomes were less commonly investigated. There was a lack of studies using longitudinal data investigating mental health of migrants that go beyond descriptive aims (i.e., explanatory and predictive), and have large nationally representative samples. Moreover, there is a need for research examining social determinants of mental health and wellbeing at the structural, family and community levels. We propose that existing nationally representative population-based surveys are used to a greater extent to study various aspects of migrants’ mental health and wellbeing.

**Supplementary Information:**

The online version contains supplementary material available at 10.1007/s10903-023-01490-5.

## Background

Switzerland is a country with the third-largest proportion of migrants in Europe (defined as population with foreign citizenship), after Luxembourg and Lichtenstein, with 2.1 million people (25% of the whole population), with majority from Eropean Union, European Free Trade Association states or the United Kingdom [1]. The migration population of Switzerland is very diverse, with large numbers of refugees from war-torn countries, such as former Yugoslavia, as well as, highly educated labour migration mainly from European countries [1]. The war in Ukraine and global warming are likely to lead to increased migration in near future. Hence, the United Nations 2030 Agenda for Sustainable Development calls for actions to build equitable and inclusive societies allowing the migrant population to fulfil their potential [2]. Migrants have a right to health and good health is essential to work, be productive and contribute to social and economic development. Migrants tend to disproportionally suffer from mental health problems, having also greater barriers to accessing mental health services. Therefore, it is important to better understand which migrant groups and migration life trajectories are particularly vulnerable to mental health problems and why.

Systematic reviews consistently show worse levels of mental health and wellbeing among migrants than non-migrants across different countries, with depression and anxiety being the most common problems in the migrant population [3]. The most comprehensive estimates of point prevalence of depression and anxiety are 38.99% (with a 95% confidence interval ranging from 27 to 51%) and 27.31% (6–58%), respectively [4]. Migrant workers also tend to experience other issues such as alcohol or substance abuse and poor sleep quality to a greater extent than the general population [3].

The risk factors for poor mental health identified in international systematic reviews are similar to the ones found in the general population, however, migrants may be affected by them to a greater extent. These included poor physical health, family history of psychiatric disorder, poor coping skills, workplace stressors, poor working conditions, limited access to healthcare, duration of residence, living conditions and limited social support [4]. Factors that were more unique to migrants include discrimination based on origin, and, partially because of that, employment in jobs for which they are overqualified or reliance on precarious employment [5]. However, it is important to emphasise that migrant population is highly diverse in Switzerland. It ranges from, mainly European, highly qualified, migrant workers, to the most vulnerable populations such as asylum seekers or refugees. Switzerland is also home to an estimated 90,000 undocumented migrants who live in precarious conditions [6]. Hence, the social determinants of mental health and wellbeing are likely to be very different in these highly heterogenous groups. One of the challenges of research on migrants’ mental health is to ensure that the available data capture the diversity of migrant population, providing generalisable information. Somewhat unexpectedly, the systematic reviews cited here did not identify any studies conducted in Switzerland after 2015. This is despite Switzerland having several representative population-based datasets with a large proportion of the migrant population. There are several potential reasons for this omission, other than simply a lack of research conducted in this context in Switzerland. First, Switzerland is a multi-lingual country, with some research potentially being published in a non-English language. Second, a lot of descriptive information about major social issues, such as migration, is provided by the Federal Statistical Office (FSO), rather than journal publications. This is in the format of online reports, which may not be picked up by academic search engines or Google Scholar. Thirdly, the definition of migrant population might be often too narrow to capture Swiss studies. Namely, a lot of studies define migrants  as, for instance, residents of a given country without citizenship. However, in Switzerland, the definition of migration, from the academic and policy perspective, tends to be wider, including ‘population with a migration background’, which comprises not only the nationality and country of birth of individuals but also that of their parents [1]. Such a conceptualisation is based on the international recommendations by the United Nations Economic Commission for Europe and is in line with the topology proposed by the Federal Statistical Office [1, 7]. The main limitation of the literature identified by these systematic reviews was a reliance on cross-sectional studies. This is not necessarily a limitation for descriptive studies, which aim to estimate the prevalence of mental health problems. However, a cross-sectional design is inadequate for predictive and explanatory aims.

There is great potential in the observational data in Switzerland, for instance, the Swiss Household Panel, to study mental health of migrants. The strength of the Swiss data in the European context is that it includes a relatively large number of migrants, representative of the migrant population. This is at odds with, for example, datasets in Great Britain, where the observational studies tend to include mainly white, nationally born participants.

Hence, the overarching aim of this review is to describe and map the current evidence on mental health of the population with migrant background in Switzerland and to identify knowledge gaps in the area. We aim to review three types of studies investigating mental health of the population with migrant background in Switzerland, using population-based and migrant-specific datasets: descriptive, predictive and explanatory [8]. Descriptive studies assess mental health of migrants, compare their mental health to non-migrant or general population or identify subgroups of migrants with particularly good or poor mental health. For instance, they may uncover whether prevalence of mental health problems varies according to a region of origin, sex, or socioeconomic group. Predictive studies aim to estimate individual risk of having poor mental health among migrant groups, that is, they help to identify individuals at increased risk of mental health problems. This could inform more targeted interventions, for instance, focused on the most vulnerable groups. However, these studies cannot tell us anything about mechanisms that translate migration into poorer mental health, as they do not consider the causal structure of the relationship between migration and mental health. Such mechanisms can be uncovered by explanatory studies, which try to answer questions related to how or why migration is associated with worse mental health outcomes. As migration cannot be randomised, a potential intervention can be identified through carefully designed observational studies.

## Method

The scoping review was deemed to be relevant for the purpose of our study, which was to describe the available evidence in the field of migrants’ mental health in Switzerland and identify knowledge gaps to inform future research [9]. As recommended, the design of this scoping review follows five stages: (1) identifying the research question, (2) identifying relevant studies, (3) study selection, (4) charting the data, (5) collating, summarising and reporting the results [10]. The protocol for the review was not pre-registered, as we aimed to identify and map relevant studies, rather than report their findings or assess their quality. Hence, there was no bias that a protocol could help to minimise, for instance, an elevated risk of false positive results.

### Identifying the Research Question

Our research questions were: What is known from the existing quantitative evidence about the mental health of the population with migrant background living in Switzerland? What are the research gaps that can be addressed with existing secondary datasets in Switzerland?

### Identifying Relevant Literature

The potentially relevant studies were first identified through a search in electronic databases. The key concepts of the search were ‘mental health and wellbeing’, ‘migration’ and ‘Switzerland’. The terms for each of these concepts were combined with OR and the concepts were combined with AND Boolean operators. The search was conducted in Ovid MEDLINE and APA PsycInfo, using Ovid platform, on the 4th of October 2022 with date limits of publication 2015–4th week of September 2022 (see eTable 1 for the exact search strategy). The included manuscripts were published in 2015–2022, however the data used in these studies might have been collected earlier. One study was published twice, in 2014 (in English) and 2016 (in German: Forum der Psychoanalyse: Zeitschrift fur klinische Theorie & Praxis. Vol.32(2), 2016, pp. 135–149) [11]. Our search picked up the 2016 manuscript, but as we found an English version published two years earlier, we decided to keep the study in.

The search resulted in a total of 1862 potentially relevant studies. In addition, we searched (1) reference lists of included studies, (2) lists of studies conducted by Swiss research organisations (Federal Statistical Office, LIVES Centre, Swiss Foundation for Research in Social Sciences), (3) relevant academic journals (Swiss medical weekly, Journal of immigrant and minority health, Ethnicity and health), (4) Google Scholar.

### Study Selection

**Fig. 1:**
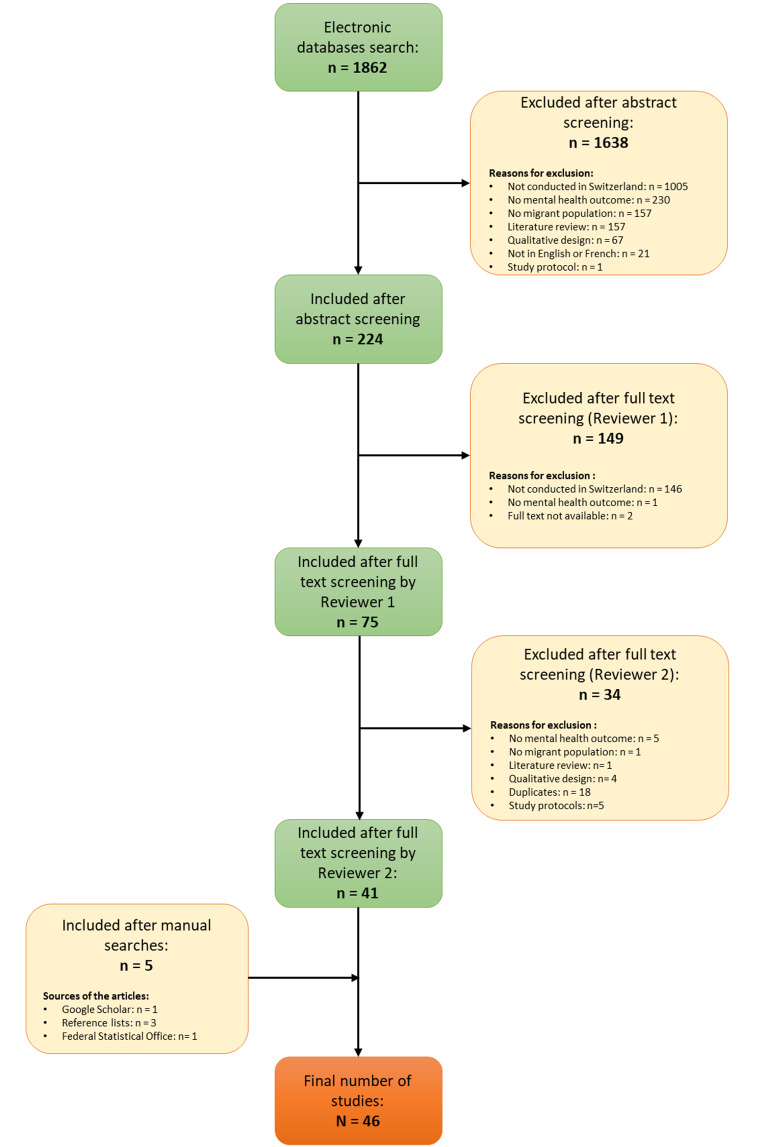
Flow diagram

The study selection is graphically presented by the flow diagram (Fig. 1). The abstracts and titles of 1862 potentially relevant publications, identified through electronic databases search, were screened for exclusion criteria by the first reviewer (NW), which were (1) qualitative design, (2) population with migrant background not included, (3) mental health or wellbeing outcome not included, (4) not published in English or French, (5) not conducted in Switzerland, (6) published before 2015 (see Table 1 for an explanation of each criterion). Any uncertainty about whether a given study should be excluded was discussed by the first and second reviewers (NV and DG). This resulted in narrowing the number of potentially relevant studies to 224. The full texts of these publications were retrieved and screened again, following a similar process as above, by the first reviewer (NV) who selected 75 potentially relevant texts. After screening by the second reviewer (DG), 41 studies were included. The manual searches resulted in adding five more studies. In total, 46 studies were included in this review.

**Table 1:** Inclusion and exclusion criteria used to identify relevant studies

Exclusion criteria	Inclusion criteria	Explanation
Qualitative design	Quantitative design	We aimed to identify gaps in quantitative research.
Population with migrant background not included	Population with migrant background included	This includes not only residents who do not hold Swiss nationality (first generation), but also those who were not born in Switzerland or whose at least one of the parents is of foreign origin (second generation). We also included refugees, asylum seekers or any other potentially temporary migrant groups.
Mental health or wellbeing outcome not included	Mental health or wellbeing outcome included	We were interested in both mental health and wellbeing outcomes, as they may be equally important for the functioning of the migrant population.
Not published in English or French	Published in English or French	These two languages were used by the team members.
Not conducted in Switzerland	Conducted in Switzerland	The focus of the review was on the Swiss context.
Published before 2015	Published in 2015 or later	The focus of the study was on relatively recent evidence to capture current migration dynamics.

### Charting the Data

The data were extracted from all included studies by the first reviewer (NV), with 20% of the studies also reviewed independently by the second reviewer for the comparison in consistency in extracted information. Any discrepancies were resolved by discussion. The information was extracted and organised using a pre-specified form, created in Microsoft Excel (see eTable 2). The studies were classified into descriptive, predictive or explanatory [8].

The social determinants examined by the studies, for instance as predictors of mental health among migrants, were classified according to the conceptual framework for public mental health, recently developed by Dykxhoorn and colleagues [12]. This comprehensive framework proposes 55 key determinants organised into four levels: individual (e.g., sociodemographic, physical health), family (i.e., family structure and dynamics), community (i.e., social, geographical and physical environment), and structural (e.g., norms, rights, government and political) [12].

### Collating, Summarising and Reporting the Results

As typically in scoping reviews, we descriptively present the key characteristics of the collated evidence, with the focus on mental health of the migrant population in Switzerland. We use evidence maps to visually summarise research characteristics, which helps to identify potential gaps in the literature [13].

## Results

### Characteristics of the Included Studies

**Fig. 2:**
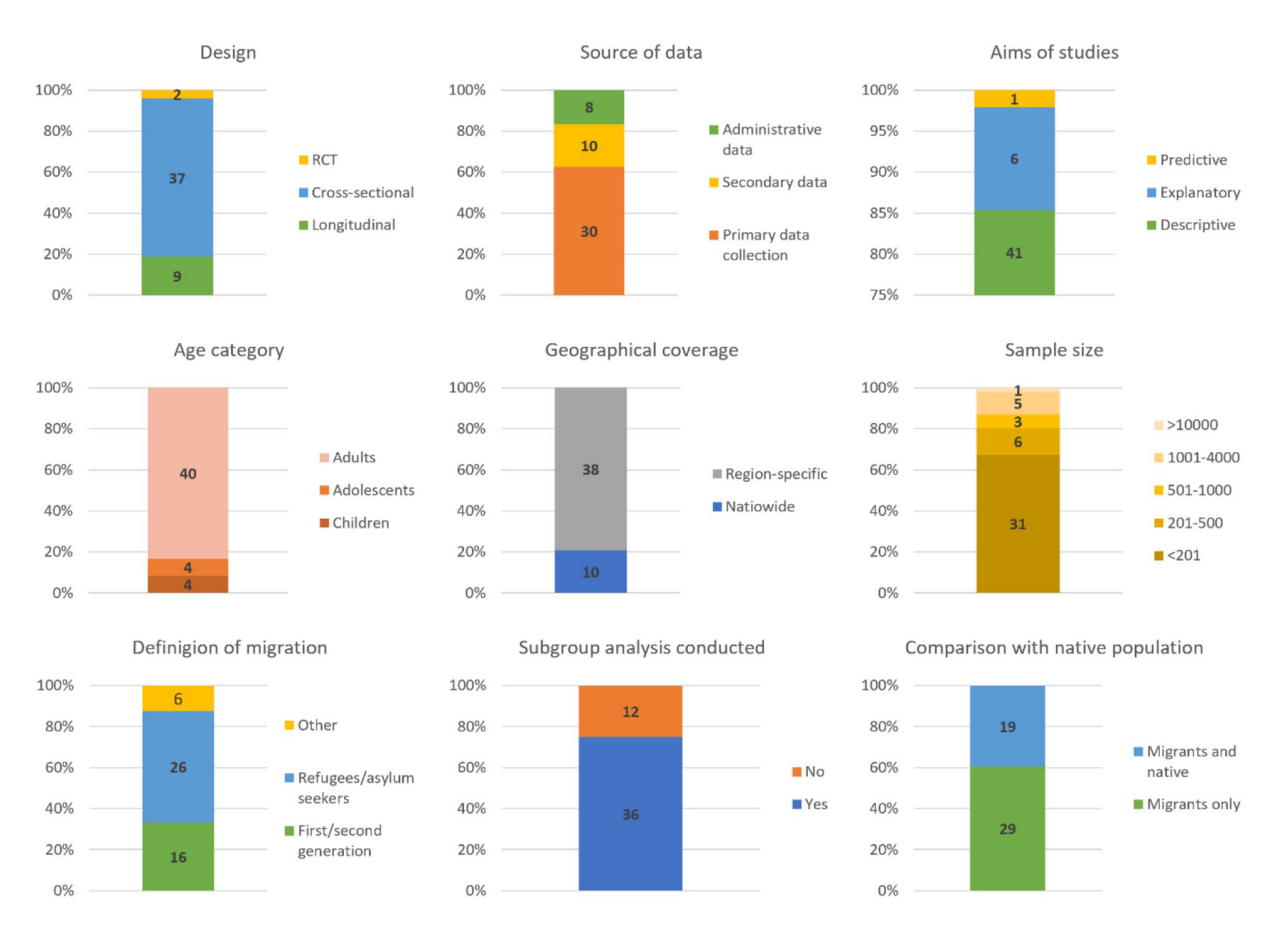
Aggregated characteristics of included studies (characteristics of individual studies can be found in eTable 3)

The aggregated characteristics of the included studies can be found in Fig. 2 and the characteristics of individual studies in eTable 2. The majority of studies used cross-sectional design (78.3%, n = 36) and their aims were descriptive (84.8%, n = 39). Most commonly researchers would collect the data for the study (referred to as ‘primary data collection’; 63.0%, n = 29), with others using existing secondary (19.6%, n = 9) or use administrative information (17.4%, n = 8). The studies relying on primary data collection would typically target selective, hard-to-reach, migrant groups, such as refugees or asylum seekers [11, 14–20]. Hence, most included studies had a sample size of under 200 participants (65.2%, n = 30) and covered narrow geographic areas such as individual cities or cantons (78.3%, n = 36).

Among nine studies that used secondary data, the datasets included the Swiss Health Survey (n = 2) [21, 22], the Swiss Household Panel (n = 1) [23], Swiss Youth Mental Health Literacy and Stigma Survey (n = 1) [24], Swiss Labour Force Survey (n = 1) [25], Gesundheitsmonitoring der Migrationsbevölkerung in der Schweiz (n = 1) [26], Swiss Multicentric Adolescent Survey on Health (n = 1) [27], two studies did not specify the sources of the secondary data [20, 28]. Nine studies used administrative data, taking advantage of medical electronic records available within hospital statistics [15, 18, 29–34]. The basic characteristics of these studies varied widely (e.g., age category, sample size, mental health outcomes; see eTable 2 for details). However, they were all limited to narrow geographical regions and all but one of them were cross-sectional [32].

Majority of the included studies comprised mainly adults (84.8%, n = 39), with a few being focused on children (6.5%, n = 3) or adolescents (8.7%, n = 4) only. About half of the studies included refugees or asylum seekers (52.2%, n = 24), one-third included first or second-generation migrants (32.6%, n = 15), and the rest comprised a combination of various migrant groups (15.2%, n = 7) – e.g., undocumented or with unspecified migration background. Most studies made comparisons of migration subgroups (76.1%, n = 35). For instance, the researchers compared first and second-generation migrants [27] or undocumented and regularised migrants [23].

### Social Determinants and Mental Health – Evidence map

**Fig. 3:**
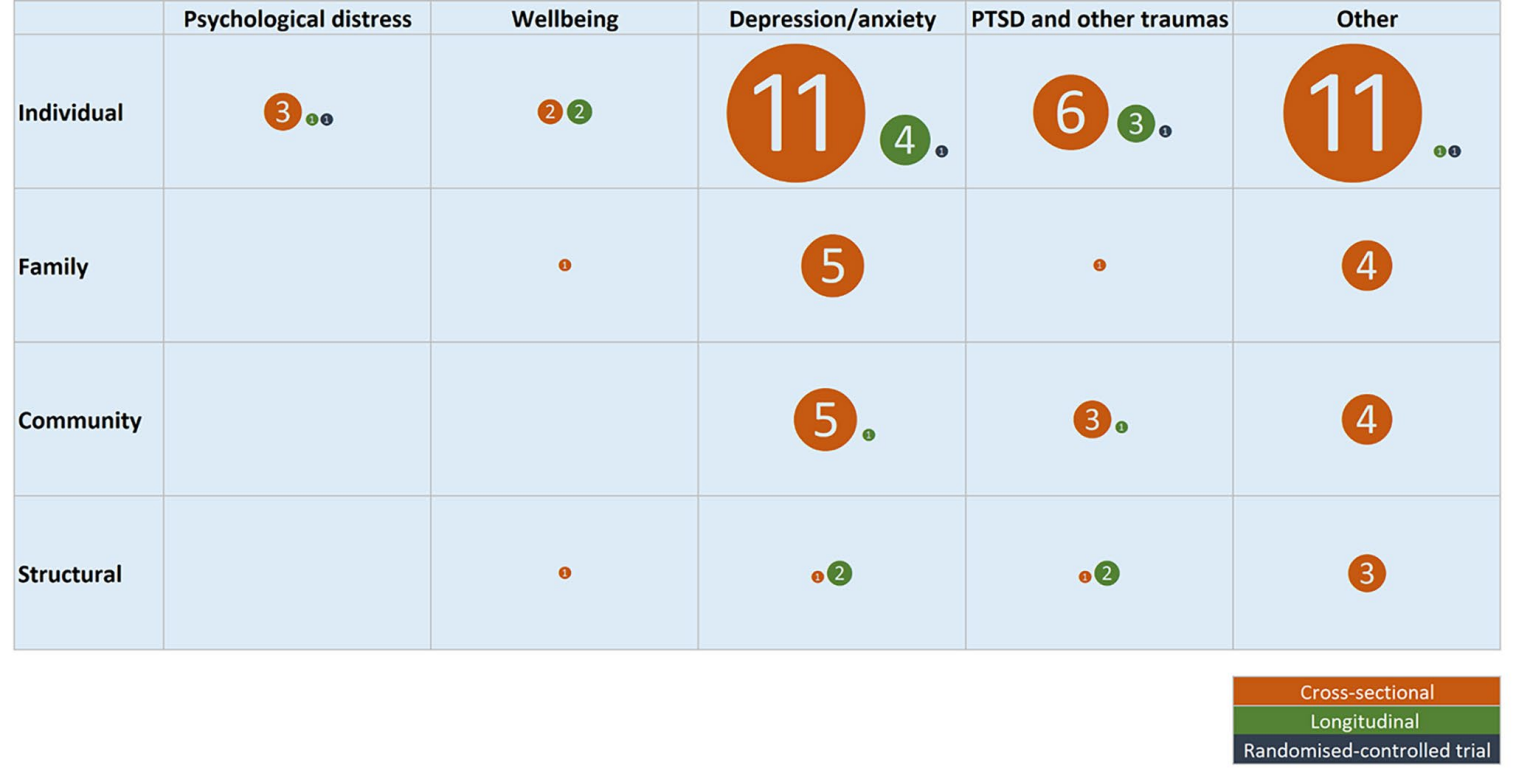
The evidence map of social determinants and mental health outcomes studied in migrant population in Switzerland – in 2015–2022

Figure 3 provides a visual representation of the distribution of social determinants and mental health outcomes examined by the included studies. More details regarding individual studies can be found in eTable 2. Most studies examined mental health or wellbeing of the population with migrant background in the context of social determinants (69.6%, n = 32). Over half of these studies included more than one type of social determinant (e.g., individual and family) (53.2%, n = 17). The most examined social determinants were at the individual level, with nearly all studies having one determinant from that group (96.9%, n = 31). The determinants at the family, community and structural levels were included in a similar number of studies (family: 28.2%, n = 9; community: 25.0%, n = 8; structural: 21.9%, n = 7). The social determinants were mainly examined as exposures (‘independent variables’) (78.1%, n = 25), with other studies defining them as effect modifiers (12.5%, n = 4), mediators (6.3%, n = 2) or a combination of exposures and mediators (3.1%, n = 1).

Out of 46 included studies, 32.6% (n = 15) included depression or anxiety, 21.7% (n = 10) PTSD and other traumas. Other outcomes were less commonly examined: psychological distress (10.9%, n = 5), wellbeing (8.7%, n = 4), mental healthcare utilisation (6.5%, n = 3). A large proportion of studies used various other outcomes (28.3%, n = 13), such as externalising problems [35], or any psychiatric disorder [15, 30–32, 36].

The most visible gap in the current evidence is a dearth of studies using longitudinal design (Fig. 3). Moreover, structural social determinants have been rarely investigated across any mental health or wellbeing outcomes, whereas family and community factors have been virtually not examined in the context of psychological distress or wellbeing.

## Discussion

The key strength of the research on mental health and wellbeing of populations with migrant background in Switzerland is relatively rich evidence on asylum seekers and refugees, typically hard-to-reach groups. However, these studies tend to rely on a cross-sectional design, small samples or narrow geographical regions.

The key evidence gaps include a dearth of studies with a longitudinal design, going beyond descriptive aims (i.e., explanatory and predictive), and with large nationally representative samples. Moreover, there is a need for research examining structural social determinants of mental health and wellbeing as well as family and community factors in the context of psychological distress and wellbeing.

### Research Recommendations

In this section, we provide recommendations for how the aforementioned evidence gaps can be addressed using existing secondary datasets and administrative data, which have been underused in the Swiss context. In Table 2, we give brief information on major population-based datasets in Switzerland, which can be used to address the identified evidence gaps.

**Table 2:** The key characteristics of secondary databases in Switzerland that include population with migrant population

Dataset	Design (sample size)	Period (age)	Migrant population	Mental health measures	Social determinants
Swiss Household Panel^1^	Longitudinal	Annually: 1999–2023 (0-103)	First/second generation	Psychological distress/wellbeing	Individual/family/community/ structural
Families and Generations Survey^2^	Repeated cross-sectional	2013, 2018, 2023 (15–79)	Year of immigration	Psychological distress/wellbeing	Individual/family/community/ structural
Swiss Health Survey^3^	Repeated cross-sectional	Every 5 years: 1992–2022 (> 15)	First/second generation	Psychological distress/depression	Individual/structural
Swiss Labour Force Survey^4^	Longitudinal	Annually: 1991–2022 (> 15)	Foreign nationality	Long-term activity limitation	Individual/family/community/ structural
European Social Survey^5^	Longitudinal	Biennially 2002–2022 (> 15)	Foreign nationality	Psychological distress/wellbeing	Individual/family/community/ structural
Survey of Health, Ageing and Retirement in Europe^6^	Longitudinal	Annually 2012–2022 (≥ 50)	Country of birth, year of immigration	Depression/anxiety/psychological distress/wellbeing	Individual/family/community/ structural
Statistics on Income and Living Conditions^7^	Longitudinal	Annually: 2016–2021 (> 15)	Foreign nationality	Wellbeing	Individual/family/community/ structural

More information:

^1^ https://forscenter.ch/projects/swiss-household-panel/

^2^ https://www.bfs.admin.ch/bfs/en/home/statistics/population/surveys/efg.html

^3^ https://www.bfs.admin.ch/bfs/fr/home/statistiques/sante/enquetes/sgb.html

^4^ https://www.bfs.admin.ch/bfs/en/home/statistics/work-income/surveys/slfs.html

^5^ https://www.europeansocialsurvey.org/

^6^ https://www.elsa-project.ac.uk/

^7^ https://www.bfs.admin.ch/bfs/en/home/statistics/economic-social-situation-population/surveys/silc.html

Despite numerous existing descriptive studies, there is still a need for country-wide research, which would provide representative prevalence or incidence of common mental health problems. For instance, the Families and Generations Survey and Swiss Health Survey could be used to provide information on the potential differences between Swiss and foreign populations in psychological distress, depression and wellbeing. As these surveys are designed to produce representative values for cross-sections of Swiss society at regular time intervals, they can shed the light on changes over time in mental health and wellbeing across various migrant groups, e.g., according to their demographic characteristics.

The longitudinal datasets, such as the Swiss Household Panel or European Social Survey (see Table 2), are best suited to conduct predictive (e.g., who is at a higher risk of future mental health problems?) or explanatory studies (e.g., why certain groups are at a higher risk of mental health problems?). These surveys include a wide range of social determinants, at the individual, family, community and structural levels – hence, can expand on the current research mainly focusing on individual social determinants. Moreover, they collect information on psychological distress and wellbeing, which have been particularly understudied. The Swiss longitudinal surveys tend to include geographical identifiers, which allow for studying the impact of structural determinants, such as unemployment or provision of mental health services, at the municipal or cantonal level.

The existing datasets share certain strengths and limitations. They have large sample sizes, are representative of Swiss society, including both native and migrant populations, and have a plethora of various social determinants. The longitudinal studies allow for building complex statistical models, in which the temporal relationships between variables can be explored, potentially uncovering causal structures between migration and mental health or wellbeing. However, these datasets tend to rely on self-reported indicators of psychological distress or wellbeing, rather than clinical information about one’s mental health.

The migrant populations in existing databases are often limited to foreign nationals, who tend to be economic migrants. This is a dominating migration group in Switzerland, with nearly 2 million persons, constituting vast majority of overall migrant population and about 27% of the working-age population in Switzerland [1]. Despite this population being relatively well off, it is still likely to be disadvantaged compared to native-born individuals [40]. The employment rate among economic migrants is still below that of native-born and economic migrants live in greater uncertainty, to some extent due to being dependent on work permits [40]. Also, labour migrants, mainly from countries outside the Organisation for Economic Co-operation and Development, are often underemployed and migrant mothers have limited access to active labour market policy tools [37]. Overall, this makes migrant workers susceptible to mental health and wellbeing problems. Due to the large size of this population, it is important for public health to understand how mental health of this population can be protected. The available large population-based datasets are well-suited to study this population, and the findings are likely to be highly generalisable to other European countries with a sizeable proportion of migrants in the labour force.

Unfortunately, the most vulnerable individuals, such as refugees or asylum seekers are not included in these surveys. These migrant groups, however, are often targeted by primary surveys, as found by our review. This shows that there is substantial interest in Switzerland in mental health and wellbeing of this population. However, these primary surveys may not be representative for vulnerable migrant populations, as they are hard to recruit and follow over time. Moreover, small sample size of the existing studies limits their usefulness in more complex statistical analyses. However, combining and harmonising these datasets could help to obtain larger sample sizes and improve their generalisability. Our study could help to identify research groups that hold access to these data and could facilitate the efforts to maximise their use. But it has be highlighted that aggregating collected data is a challenging process due to multiple methodological differences in the recruitment of participants or used surveys.

Finally, large population-based surveys have some common methodological challenges, such as widespread, non-random, missing information due to dropout or non-response [38]. Potential biases due to missing information can be mitigated to some extent by using various imputation methods [39].

### Limitations of the Review

There are several limitations of this review. Firstly, we only included studies published in English and French. Other languages, such as German and Italian are also spoken in Switzerland, hence local reports written in these languages might have been missed. Peer-reviewed publications and national reports were unlikely to be written only in either of these languages, however it is possible that we might have omitted small-scale studies, conducted on a regional level We do not believe that the overall interpretation of the identified evidence gaps would change drastically (e.g., that family-level social determinants have been rarely examined).

Secondly, our search was limited to two major electronic databases – Ovid MEDLINE and APA PsycInfo. As this search resulted in many studies that did not meet inclusion criteria, we devoted more resources to hand searches of relevant journals and publications list of national research organisations. We found only a few additional studies (n = 5) through a hand search, hence providing evidence for the main search being comprehensive. Future reviews could also consider qualitative evidence, which would be particularly useful in theory generation for predictive and explanatory studies in the context of wellbeing and mental health of migrants.

## Conclusion

There is a relatively large amount of evidence on mental health and wellbeing of asylum seekers and refugees, hard-to-reach groups. However, these studies tend to rely on a cross-sectional design, small samples or narrow geographical regions. There is a lack of studies using longitudinal data, going beyond descriptive aims (i.e., explanatory and predictive), with large nationally representative samples. Moreover, there is a need for research examining structural social determinants of mental health and wellbeing, as well as family and community factors in the context of psychological distress and wellbeing. We propose that data already collected through large, nationally representative population-based surveys are used to a greater extent to examine various aspects of migrants’ mental health and wellbeing.

### Electronic Supplementary Material

Below is the link to the electronic supplementary material.

Supplementary Material 1

Supplementary Material 2

## Declarations

The authors report no declarations of interest.

## References

[1] Office FS (2020). A Panorama of Swiss Society 2020: Migration—Integration—Participation.

[2] Nations U (2015). Transforming our world: the 2030 agenda for Sustainable Development.

[3] Mucci N, Traversini V, Giorgi G, Tommasi E, De Sio S, Arcangeli G. Migr Workers Psychol Health: Syst Rev Sustain 2020;12(120).

[4] Hasan SI, Yee A, Rinaldi A, Azham AA, Mohd Hairi F, Amer Nordin AS (2021). Prevalence of common mental health issues among migrant workers: a systematic review and meta-analysis. PLoS ONE.

[5] Hargreaves S, Rustage K, Nellums LB, McAlpine A, Pocock N, Devakumar D (2019). Occupational health outcomes among international migrant workers: a systematic review and meta-analysis. The Lancet Global Health.

[6] (FOPH) FOoPH. Healthcare provision for undocumented migrants 2023 [Available from: https://www.bag.admin.ch/bag/en/home/strategie-und-politik/nationale-gesundheitsstrategien/gesundheitliche-chancengleichheit/chancengleichheit-in-der-gesundheitsversorgung/gesundheitsversorgung-der-sans-papiers.html.

[7] (UNECE) UNECfE. Conference of European Statisticians Recommendations for the 2020 Censuses of Population and Housing. New York and Geneva: United Nations; 2015.

[8] Shmueli G (2010). To explain or to predict?. Stat Sci.

[9] Munn Z, Peters MDJ, Stern C, Tufanaru C, McArthur A, Aromataris E (2018). Systematic review or scoping review? Guidance for authors when choosing between a systematic or scoping review approach. BMC Med Res Methodol.

[10] Arksey H, O’Malley L (2005). Scoping studies: towards a methodological framework. Int J Soc Res Methodol.

[11] Heeren M, Wittmann L, Ehlert U, Schnyder U, Maier T, Müller J (2014). Psychopathology and resident status - comparing asylum seekers, refugees, illegal migrants, labor migrants, and residents. Compr Psychiatr.

[12] Dykxhoorn J, Fischer L, Bayliss B, Brayne C, Crosby L, Galvin B (2022). Conceptualising public mental health: development of a conceptual framework for public mental health. BMC Public Health.

[13] Miake-Lye IM, Hempel S, Shanman R, Shekelle PG. What is an evidence map? A systematic review of published evidence maps and their definitions, methods, and products. Syst Rev. 2016;5(28). 10.1186/s13643-016-0204-x PMC4750281 26864942

[14] Benz T, Lehmann S, Brioschi R, Elfering A, Aeschlimann A, Angst F (2019). Comparison of short- and mid-term outcomes of italian- and german-speaking patients after an interdisciplinary pain management programme in Switzerland: a prospective cohort study. J Rehabilitation Med ORIGINAL Rep J Rehabil Med.

[15] Buser S, Brandenberger J, Gmünder M, Pohl C, Ritz N (2021). Asylum-seeking children with Medical Complexity and Rare Diseases in a Tertiary Hospital in Switzerland. J Immigr Minor Health.

[16] Chernet A, Probst-Hensch N, Sydow V, Paris DH, Labhardt ND. Mental health and resilience among Eritrean refugees at arrival and one-year post-registration in Switzerland: a cohort study. BMC Res Notes. 2021;14(1). 10.1186/s13104-021-05695-5 PMC8299667 34294120

[17] Harju E, Roser K, Dehler S, Michel G (2018). Health-related quality of life in adolescent and young adult cancer survivors. Support Care Cancer.

[18] Jackson Y, Paignon A, Wolff H, Delicado N. Health of undocumented migrants in primary care in Switzerland. PLoS ONE. 2018;13(7). 10.1371/journal.pone.0201313 PMC6063438 30052674

[19] Lacour O, Morina N, Spaaij J, Nickerson A, Schnyder U, von Känel R et al. Prolonged Grief Disorder Among Refugees in Psychological Treatment—Association With Self-Efficacy and Emotion Regulation. Front Psychiatry. 2020;11. 10.3389/fpsyt.2020.00526 PMC7291948 32581893

[20] Le L, Morina N, Schnyder U, Schick M, Bryant RA, Nickerson A (2018). The effects of perceived torture controllability on symptom severity of posttraumatic stress, depression and anger in refugees and asylum seekers: a path analysis. Psychiatry Res.

[21] Storni M (2020). Enquête suisse sur la santé 2017. État de santé de la population issue de la migration.

[22] Schneeberger AR, Seixas A, Schweinfurth N, Lang UE, Cajochen C, Bux DA et al. Differences in insomnia symptoms between immigrants and non-immigrants in Switzerland attributed to emotional distress: Analysis of the swiss health survey. Int J Environ Res Public Health. 2019;16(2). 10.3390/ijerph16020289 PMC6352062 30669632

[23] Burton-Jeangros C, Duvoisin A, Consoli L, Fakhoury J, Jackson Y. The well-being of newly regularized migrant workers: Determinants of their satisfaction with life as compared to undocumented migrant workers and regular local residents. Comp Migration Stud. 2021;9(1). 10.1186/s40878-021-00244-2 PMC8550693 34722159

[24] Dey M, Castro RP, Jorm AF, Marti L, Schaub MP, Mackinnon A. Stigmatizing attitudes of Swiss youth towards peers with mental disorders. PLoS ONE. 2020;15(7 July). 10.1371/journal.pone.0235034 PMC7380889 32706786

[25] Rellstab S, Pecoraro M, Holly A, Wanner P, Renard K. The Migrant Health Gap and the Role of Labour Market Status: Evidence from Switzerland. 2016.

[26] Moussa JS, Pecoraro M, Ruedin D, Houmard S, Khanlou N, Pilkington FB (2015). The gender gap in Mental Health: immigrants in Switzerland. Women’s Mental Health: Resistance and Resilience in Community and Society.

[27] Vazsonyi AT, Mikuska J, Gassova Z (2017). Revisiting the immigrant paradox: suicidal ideations and suicide attempts among immigrant and non-immigrant adolescents. J Adolesc.

[28] Weber S, Landolt MA, Maier T, Mohler-Kuo M, Schnyder U, Jud A (2017). Psychotherapeutic care for sexually-victimized children – do service providers meet the need? Multilevel analysis. Child Youth Serv Rev.

[29] Ambrosetti J, Macheret L, Folliet A, Wullschleger A, Amerio A, Aguglia A et al. Psychiatric emergency admissions during and after COVID-19 lockdown: short-term impact and long-term implications on mental health. BMC Psychiatry. 2021;21(1). 10.1186/s12888-021-03469-8 PMC8464091 34560856

[30] Frizi R, Lay B, Seifritz E, Kawohl W, Habermeyer B, Roser P. Sociodemographic and Clinical Predictors of the Length of Psychiatric Inpatient Stay of Immigrants in Switzerland. Front Psychiatry. 2020;11. 10.3389/fpsyt.2020.585798 PMC7755930 33362603

[31] Gmünder M, Brandenberger J, Buser S, Pohl C, Ritz N (2020). Reasons for admission in asylum-seeking and non-asylum-seeking patients in a paediatric tertiary care centre. Swiss Med Wkly.

[32] Hotzy F, Hengartner MP, Hoff P, Jaeger M, Theodoridou A (2019). Clinical and socio-demographic characteristics associated with involuntary admissions in Switzerland between 2008 and 2016: an observational cohort study before and after implementation of the new legislation. Eur Psychiatry.

[33] Schoretsanitis G, Bhugra D, Eisenhardt S, Ricklin ME, Srivastava DS, Exadaktylos A et al. Upon rejection: Psychiatric emergencies of failed asylum seekers. Int J Environ Res Public Health. 2018;15(7). 10.3390/ijerph15071498 PMC6069106 30012985

[34] Schoretsanitis G, Eisenhardt S, Ricklin ME, Srivastava DS, Walther S, Exadaktylos A. Psychiatric emergencies of asylum seekers; descriptive analysis and comparison with immigrants of warranted residence. Int J Environ Res Public Health. 2018;15(7). 10.3390/ijerph15071300 PMC6068840 29933607

[35] Aksoy D, Favre CA, Janousch C, Ertanir B. Internalizing and Externalizing Symptoms in Adolescents With and Without Experiences of Physical Parental Violence, a Latent Profile Analysis on Violence Resilience. Front Psychol. 2022;13. 10.3389/fpsyg.2022.824543 PMC9008205 35432093

[36] Premand N, Baeriswyl-Cottin R, Gex-Fabry M, Hiller N, Framorando D, Eytan A (2018). Determinants of suicidality and of treatment modalities in a community psychiatry sample of asylum seekers. J Nerv Mental Disease.

[37] Vidal-Coso E, Steiner I, Wanner P (2019). Employment trajectories of recent immigrants in Switzerland. Migrants and expats: the Swiss Migration and mobility Nexus.

[38] Voorpostel M (2010). Attrition patterns in the Swiss Household Panel by demographic characteristics and social involvement. Attrition Swiss Journal Patterns of Sociology.

[39] Jäger S, Allhorn A, Bießmann F. A Benchmark for Data Imputation Methods. Front Big Data. 2021;4. 10.3389/fdata.2021.693674 PMC8297389 34308343

[40] Auer, D. (2018). Drivers of immigrant employment in Switzerland. PhD Thesis. Lausanne, Switzerland: University of Lausanne.

