# Supplementary material for: Mental Health and Wellbeing of Population with Migrant Background in Switzerland – a Scoping Review and Evidence Map of Quantitative Evidence
Source: J Immigr Minor Health. 2023 May 26;25(5):1108–17. doi: 10.1007/s10903-023-01490-5 (PMC10509096; doi:10.1007/s10903-023-01490-5)
Supplement: Supplementary file 1 — Supplementary Material 1 [file 10903_2023_1490_MOESM1_ESM.doc]

| Supplementary eTable 1. The search strategy. | | | |
| --- | --- | --- | --- |
| **Key words** | **Fields** | **Hits: 1862** | 04/10/22 |
| **Mental Health and wellbeing** | Abstract | emotional problem* OR mental health OR mental disorder* OR mental illness OR psychiatric disorder* OR mentally ill OR psychological distress OR depression OR depressed OR depressive OR conduct disorder OR anger OR emotional adjustment OR emotional maladjustment OR behavioural problem* OR behavioural disorder* OR internali* OR externali* OR suicid* OR psychological development OR negative affect OR life satisfaction OR positive affect OR quality of life OR wellbeing OR well-being OR happ* OR positive emotion* | |
| **AND** |  |  | |
| **Migration** | Abstract | migration OR migrant OR asylum seek* OR refugees OR expat OR immigrant OR undocumented migrant OR documented migrant OR expat OR exil* OR emigrant OR immigrant | |
| **AND** |  |  | |
| **Switzerland** | All fields | Switzerland OR Swiss OR Canton | |
| Limits: publication year 2015 – 04/01/2022  ((emotional problem* or mental health or mental disorder* or mental illness or psychiatric disorder* or mentally ill or psychological distress or depression or depressed or depressive or conduct disorder or anger or emotional adjustment or emotional maladjustment or behavioural problem* or behavioural disorder* or internali* or externali* or suicid* or psychological development or negative affect or life satisfaction or positive affect or quality of life or wellbeing or well-being or happ* or positive emotion*) and (migration or migrant or asylum seek* or refugees or expat or immigrant or undocumented migrant or documented migrant or expat or exil* or emigrant or immigrant)).ab. and (Switzerland or Swiss or Canton).af.  limit 1 to yr="2015 -Current" | | | |
